# How Nitric Oxide Increases in Diabetic Morphine Tolerated Male Rats

**Published:** 2017

**Authors:** Yassar Mortada, Khojasteh Khojasteh, Malek Zarei, Ardalan Mansouri, Masoumeh Jorjani

**Affiliations:** a *Department of Pharmacology, Schoolof Medicine, Shahid Beheshti University of Medical Sciences, Tehran, Iran. *; b *Cellular and Molecular Biology Research Center & Department of Pharmacology, school of Medicine, Shahid Beheshti University of Medical Sciences, Tehran, Iran.*

**Keywords:** Diabetes, Morphine tolerance, Nitric Oxide Synthase, Cationic Amino Acid Transporter-2, Male Rat

## Abstract

Neuropathic pain is a complication of inflammation, infection or some diseases such as diabetes. Opioids are used as a salvage therapy for neuropathic pain but tolerance restricts their use. In our previous study, we have observed an increase of Nitric Oxide in diabetes and in morphine tolerance. This study was performed to clarify the role of inducible nitric oxide synthase, iNOS, and cationic amino acid transporter-2, CAT-2, in these conditions.

Thus male rats were divided into four groups: control, diabetic, morphine tolerated, and diabetic morphine tolerated. For evaluating tolerance Hot-Plate test was achieved. Molecular study was performed by real time PCR and Western blotting techniques to compare gene and protein expressions.

Our findings showed that in diabetic animals, morphine tolerance occurred prior to non-diabetic rats. In molecular study, the expression of iNOS was increased in the spinal cord whereas the CAT-2 did not change in diabetic morphine tolerated rats.

It seems that the nitric oxide elevation in diabetic morphine tolerated state is mostly due to the increase of iNOS in male rats.

## Introduction

Neuropathic pain is a problem which is frequently seen in clinical practice and is characterized by hyperalgesia which makes it difficult to be treated by analgesics (1). There are many conditionscreating neuropathic pain including inflammation, infection, or some diseases such as diabetes. It has been shown that the production of nitric oxide (NO) which is over made by inducible nitric oxide synthase (iNOS) in these states has a significant role in the appearance of neuropathic pain (2-4). NO is synthesized from L-arginine by different subtypes of nitric oxide synthase (5) in many cell types. In physiologic conditions it has different roles in the body such as platelet aggregation, cytotoxic function of macrophages, and neurotransmission in the peripheral and central nervous system. NO is involved in both nociception (6) and morphine tolerance (7). Nitric oxide plays an important role in afferent signaling and modulating of pain through the dorsal horn of the spinal cord and brain (8).

It has been shown that in pathologic disorders such as neuropathic pain, iNOS is up-regulated in the spinal cord of rats (9). Moreover, recent studies in pharmacology have shown that iNOS inhibitors decrease hyperalgesia induced by spinal injury (2, 9, 10). 

Morphine exerts analgesia by µ-opioid receptors in the spinal cord and also in supra spinals. Nitric oxide plays a role in modulating morphine action on its receptor, which can be helpful to interfere in order to enhance the analgesic effect of morphine and also to minimize the tolerance to its effects (11). It has been reported that chronic administration of morphine increases nitric oxide synthesis in rat spinal cord (12). Another study shows that NOS activity increases in the brain at 48 and 72 h after morphine treatment (13).

It has been recently reported that deletion of the iNOS gene during the induction of inflammation, partially prevents tolerance to the anti-nociceptive effects of morphine and decreases withdrawal symptoms caused by naloxone (14). 

Furthermore, it has been reported that hyperglycemia has a role in pain perception and therefore, will change the effect of morphine and causes earlier tolerance to its analgesic affects in diabetic neuropathy, thus morphine has a low analgesic effect in this situation (15-18). On the other hand, the effect of hyperglycemia on pain threshold is conflicting and some other studies showed that effect of morphine increases in induced diabetes (19-24). 

One of the mechanisms that is known to be involved in diabetes and morphine tolerance is the overproduction of NO (25). The precursor of NO is L-arginine which is carried from the outside through the cell membranes by cationic amino acid transporters (CATs) (26, 27). Till now 5 subtypes of CATs have been introduced. The most commons are CAT-1 and CAT-2. CAT-1 is expressed constitutively in mammalian cells, while CAT-2 expression is induced during inflammation (28). Some studies show that CAT-1 expression decreases in inflammatory conditions suggesting that it may have a minimal role in arginine uptake in this situation, but CAT-2 has a greater role in L- arginine transport in these states, and the increase in CAT-2 mRNA is harmonious with rises in iNOS mRNA (29). Further molecular studies are needed adjacent to behavioral studies to clarify the role and the source of nitric oxide in diabetic neuropathy and morphine tolerance. 

Thus in this study, we evaluated the expression of iNOS and CAT-2 in the spinal cord and brain of male diabetic rats after morphine tolerance and tried to discover the possible relation between the expression levels of these factors and analgesic effect of morphine in diabetic state.

## Materials and methods

*Animals*

Twenty four male Wistar rats (180–250 g) were used. Animals were housed in a room with ambient temperature of 22 ± 2 °C, a 12-h light/dark cycle and free access to water and food. They were allowed to habituate to the housing facilities 1 week prior to the behavioral testing. The recommendations and policies of the International Association for the Study of Pain (30) and the Institutional Animal Welfare Law were considered in all procedures of experiment. All study protocols were approved by the internal deputy for animal research and the respective local government committee which is advised by an independent ethics committee in our faculty. 

The animals were divided into four groups: control, diabetic, morphine tolerated, and diabetic morphine tolerated. Each group was containing 6 rats which were used only once.

*Drug preparation*

Morphine sulfate, Alloxan and Naloxone (Sigma, U.S.A.), all were dissolved in distilled water. Morphine and Naloxone were injected intraperitoneally (IP), and Alloxan was administered via subcutaneous (SC) route under the skull′s skin between the ears. 

*Induction of experimental diabetes*

The diabetes was induced by a single injection of Alloxan (100 mg/Kg, SC). 72 h after Alloxan administration, the urinary glucose level was measured colorimetrically using a tape-test. Animals with serum glucose levels higher than 300 mg/dL were considered as the diabetic rats and below this level were omitted.

*Induction of tolerance*

For the induction of tolerance in rats, repeated doses of morphine sulfate (10 mg/Kg, IP) were administered once daily for 7 consecutive days. To assess the tolerance to morphine antinociception effect, the Hot-Plate test was used and after the occurrence of tolerance ,for confirmation, the withdrawal signs (jumping, chewing, urine and feces) were recorded for ten minutes in morphine tolerated animals by the use of Naloxone (2 mg/Kg IP) which was injected thirty minutes after the last morphine dose.

*Nociceptive testing*

The nociceptive threshold was measured by hot-plate test. Animals were placed on the hot plate (55 ± 0.5 °C) and Paw Withdrawal Latency (PWL) was recorded according to the procedure described by Eddy and Leimback in 1953 (31). The reaction time measured was either hind paw licking or the plate jumping off. The cut-off point imposed was 60 s to avoid tissue damage (32), and paw edema or redness with repeated testing was not observed. Baseline latency was determined first, and the morphine analgesia was evaluated 30 minutes after the injection of the drug every other day.

*The Control Group*

The distilled water as the vehicle of Alloxan was injected once SC in rats of control group and also IP every day as the vehicle of morphine. The nociceptive testing was also evaluated in the same manner in this group. 

*Tissue isolation*

Three rats were randomly selected in each group and after CO_2 _inhalation were sacrificed. Afterwards, the spinal cord and brain were isolated and stored at -80 ºC for further molecular study.

*RNA isolation*

For RNA extraction, the RNX plus (Sinaclon), chloroform (Merck), isopropanol (Merck) and TE buffer (10 mM Tris, pH 8.0 with HCl, 1 mM EDTA) were used throughout the Sinaclon protocol. The integrity and quality of RNA samples were assessed by spectrophotometry and horizontal electrophoresis.

*Real-time PCR analysis*

Reverse transcription was carried out using M-MuLV reverse transcriptase (Thermoscientific) from 1 µg of total RNA, according to the manufacturer’s protocol. Quantitative real-time PCR was performed to determine the expression of iNOS and CAT-2 with SYBR Green PCR master mix (Applied Biosystems, Foster City, CA, USA) using an Applied Biosystems step one instrument. Relative mRNA levels were calculated using the comparative CT method as described by the manufacturer (Applied Biosystems, Foster City, CA, USA). The house-keeping gene GAPDH was used as an internal control for normalization. The Primer sequences used for quantitative real-time PCR are listed in Table 1.

Real time was performed after cDNA synthesis by RT-PCR technique, and then the results were checked by western blotting method. 

*Western blotting*

Western blotting technique was used for evaluating the expression of proteins of interest in experimental groups and control group following sample extraction and SDS-PAGE. Spinal cord and brain tissue samples were lysed in RIPA buffer (150 mM NaCl, %1 NP-40, 50 mM Tris pH = 8.0, %1 SDS, %0.5 sodium deoxycholate, 1mM EDTA and protease inhibitor cocktail) and centrifuged at 12,000 rpm at 4 ºC for 20 min. SDS sample buffer was added to aliquots of tissue extracts. Samples were placed in a water bath at 100 ºC for 5 min. Proteins were separated by 12% SDS-PAGE. Proteins were transferred to the blot. Blots were incubated with specific primary polyclonal rabbit antibodies against iNOS (1:500 dilution) (Santa Cruz) and GAPDH (cell signaling), and primary polyclonal goat antibodies against CAT-2 (Santa Cruz) in TBS-T for 18h. Then, the blot was incubated with secondary anti-rabbit and anti-goat (1:500 dilution) (Santa Cruz) in TBS-T for 90 min separately. The iNOS and CAT-2 immune-reactive proteins were detected with advanced chemiluminescence (Enhanced Chemiluminescence, Amersham Biosciences) and exposed to a film. An image analysis system (Image j, version 1.46r) was used to measure the signal intensity of the blots.

*Statistical analysis*

All experiments were performed in triplicates, and the results were expressed as mean ± standard error of means (SEM). Statistical analyses were performed by “SPSS 18” using one-way ANOVA, followed by Tukey’s posttest. P < 0.05 was considered to be statistically significant. 

## Results

*Effect of experimental diabetes on morphine analgesia and tolerance*

**Figure 1:**
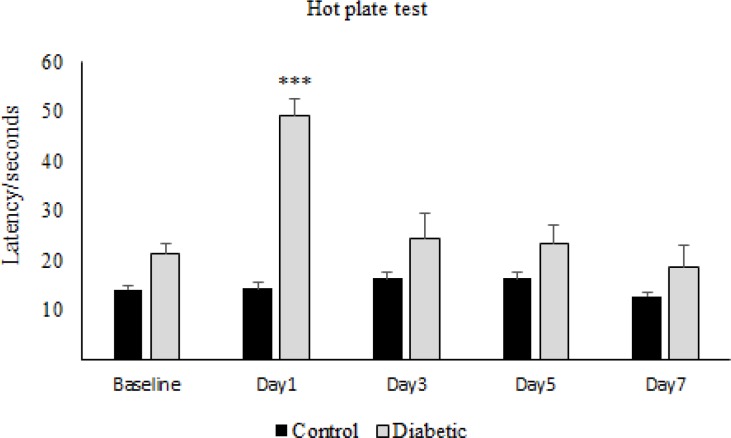
**Effect of experimental diabetes on pain threshold in rats**. The latency time at different days in control and diabetic groups was assessed. Single dose of Alloxan (100 mg/Kg, SC) was used. Data are expressed as means ± S.E.M. of 6 rats in each group. ***P < 0.001 indicates significant difference between diabetic and control group

**Figure2:**
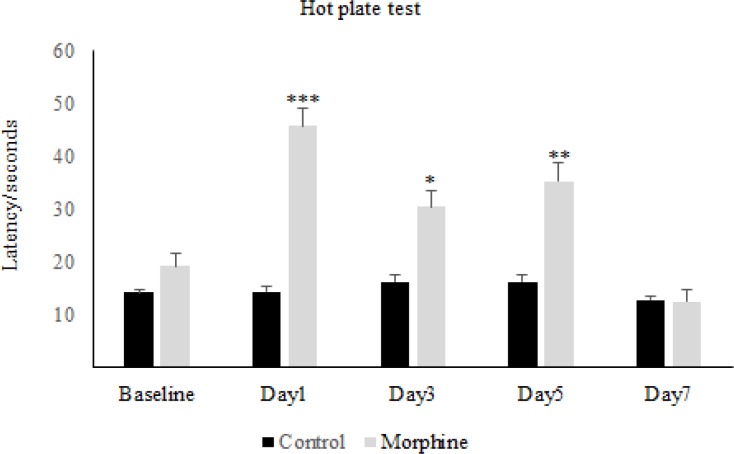
**Morphine antinociception and tolerance in rats**. The latency time at different days in morphine and control groups was investigated. Morphine sulfate (10 mg/Kg, I.P.) was injected. Data are expressed as means ± S.E.M. ***P < 0.001, **P < 0.01, *P<0.05 indicate significant difference between morphine and control group

**Figure 3:**
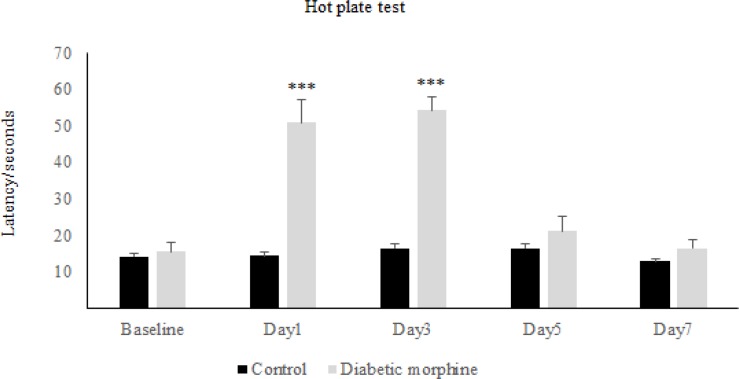
**Effect of experimental diabetes on morphine antinociception and tolerance in rats**. Pain threshold at several days in diabetic morphine and control group was measured. Morphine sulfate (10 mg/Kg, I.P.) was used. Data are expressed as means ± S.E.M. ***P < 0.0001 indicates significant difference between control and diabetic morphine groups

The results obtained from the hot-plate test has shown that in the first day of diabetes, the pain threshold in the diabetic group significantly increased compared to the control group, while on the other days of the experiment, there was not any significant difference between diabetic and control group (Figure 1). On the other hand, repeated administration of morphine showed that latency time in the morphine group increased significantly in comparison to the control group and also to the baseline, on days 1, 3, and 5 but not on day 7 (Figure 2). Whereas daily injection of morphine to the diabetic morphine tolerated group increased pain threshold significantly on days 1 and 3 in comparison to the control group and baseline and this effect was not seen in the other days of the study (Figure 3).

**Figure 4.**
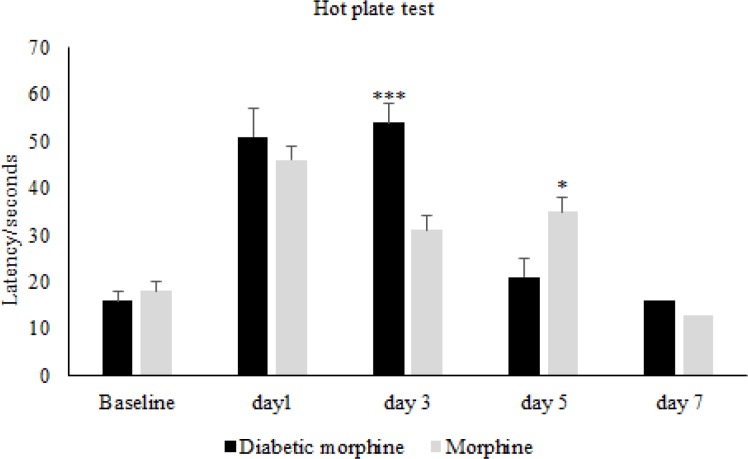
Comparison of pain threshold between morphine and diabetic morphine groups. Morphine sulfate (10 mg/Kg, I.P.) was used. Data are expressed as means ± S.E.M. ***P < 0.0001, **P < 0.01 indicate significant difference between morphine and diabetic morphine groups

**Figure 5:**
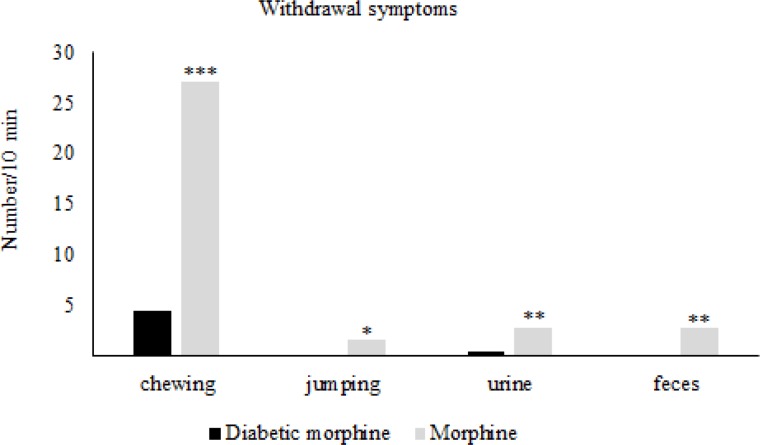
Effect of experimental diabetes on naloxone-precipitated withdrawal signs in morphine tolerated rats. Morphine sulfate (10mg/Kg, IP) and naloxone (2 mg/Kg IP, single dose) were injected. Data are expressed as means ± S.E.M. *P < 0.05, **P < 0.01, ***P < 0.001 indicate significant difference between diabetic and nondiabetic groups

**Figure 6:**
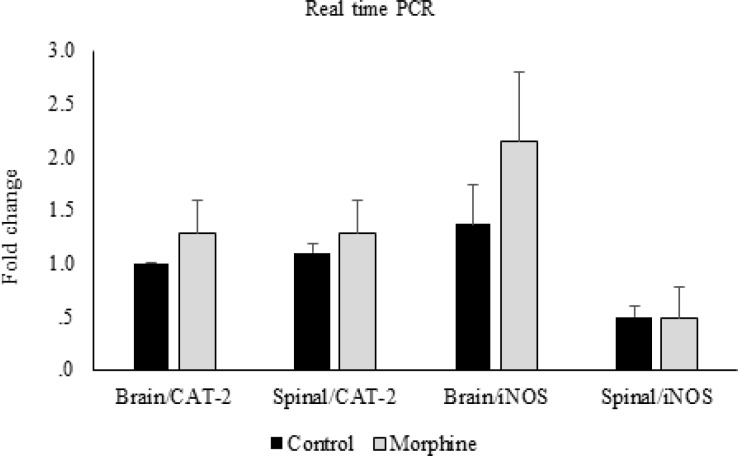
CAT-2 and iNOS gene expression. Comparison of CAT-2 and iNOS gene expression between control and morphine tolerated groups in brain and spinal cord. Data are expressed as means ± S.E.M

**Figure 7:**
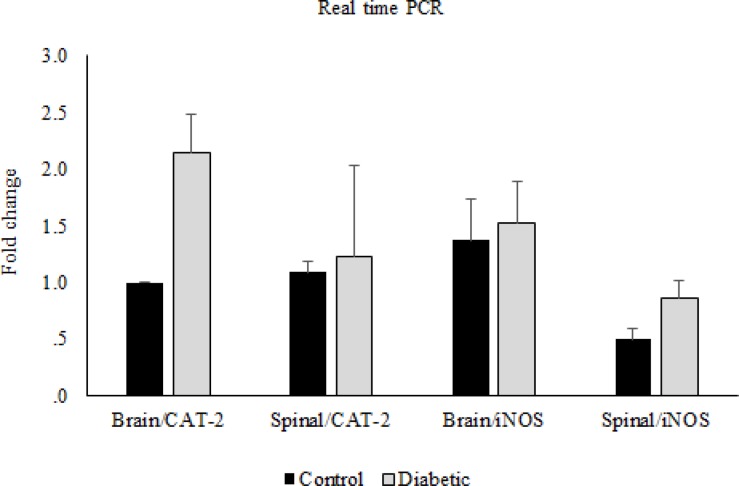
CAT-2 and iNOS gene expression. Comparison of CAT-2 and iNOS gene expression between control and diabetic groups in brain and spinal cord. Data are expressed as means ± S.E.M

**Figure 8:**
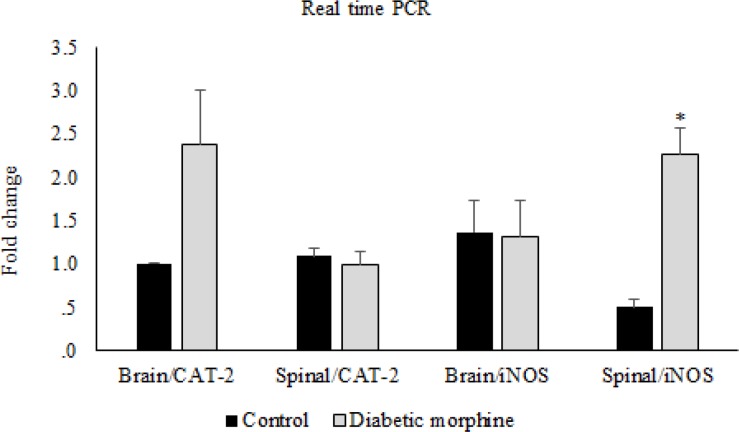
CAT-2 and iNOS gene expression. Comparison of CAT-2 and iNOS gene expression between control and diabetic morphine groups in spinal cord and brain. Data are expressed as means ± S.E.M. *P < 0.05 indicates significant increase in diabetic morphine group compared to the control group

**Figure 9:**
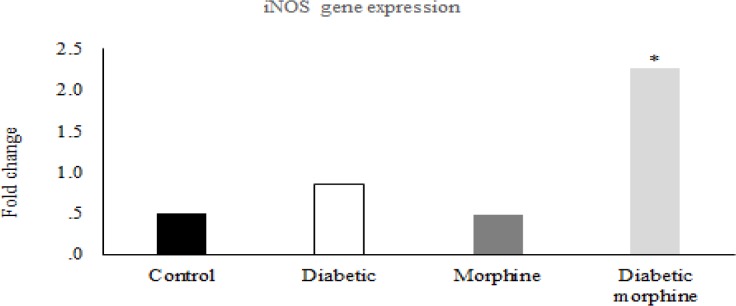
iNOS gene expression in the spinal cord. Comparison of iNOS gene expression between Control, Diabetic, Morphine and Diabetic morphine groups. Data are expressed as means ± S.E.M. *P < 0.05, indicates Significant increase in diabetic morphine group compared to the other groups

**Figure 10:**
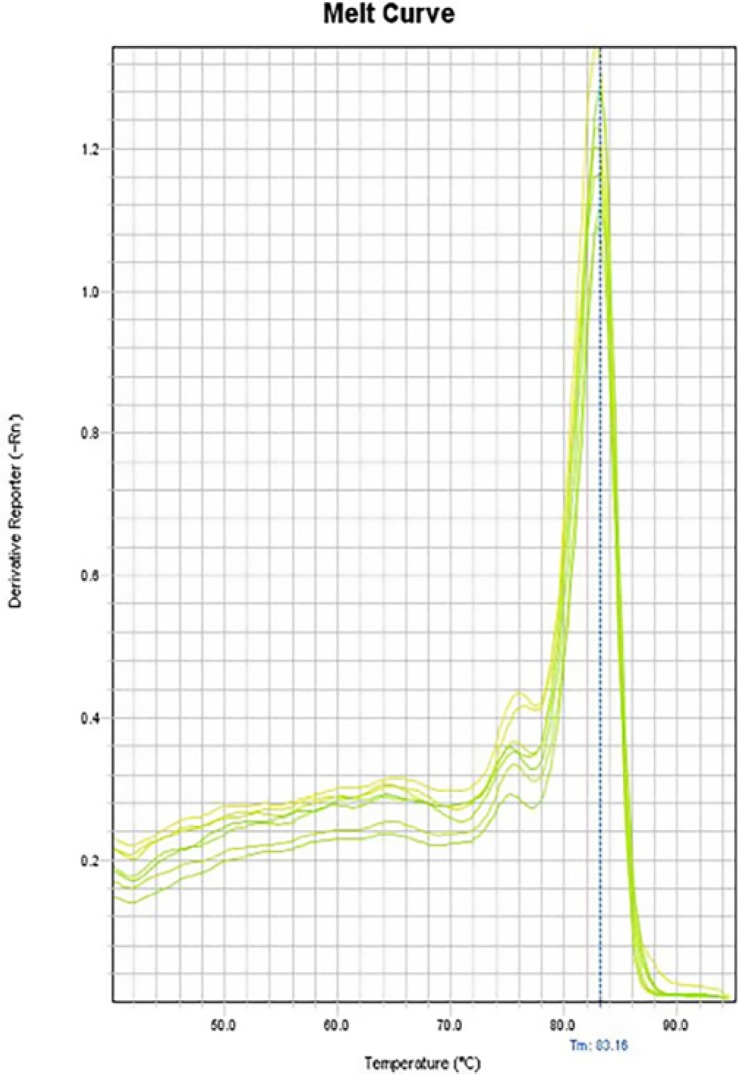
Melting curve of CAT-2

**Figure 11:**
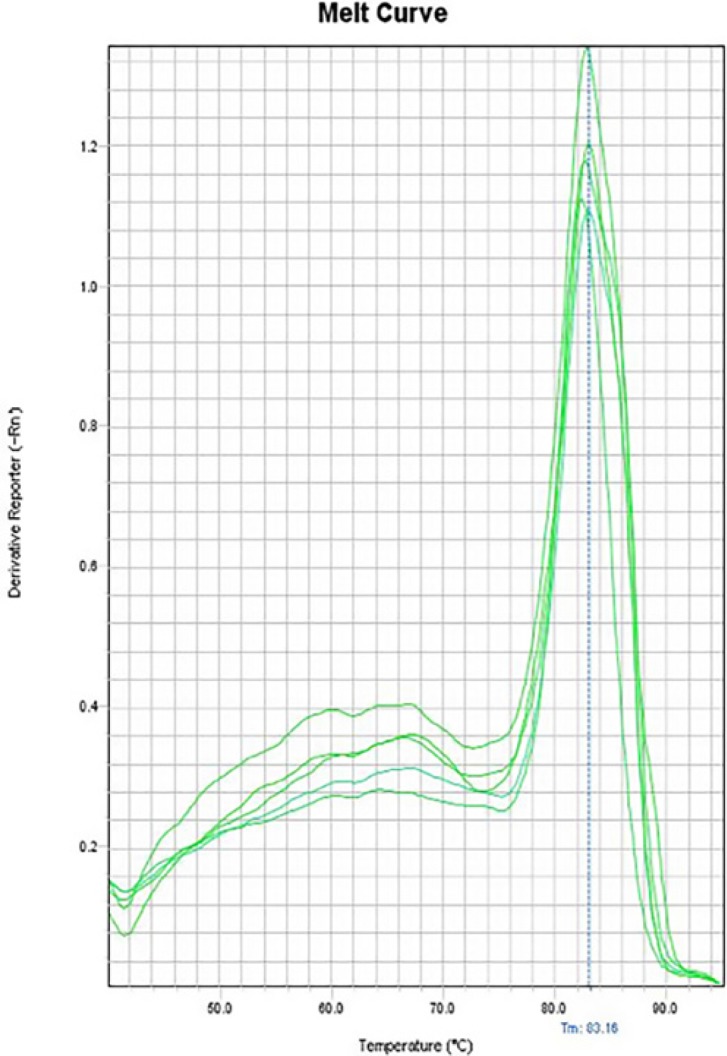
Melting curve of iNOS

**Figure 12:**
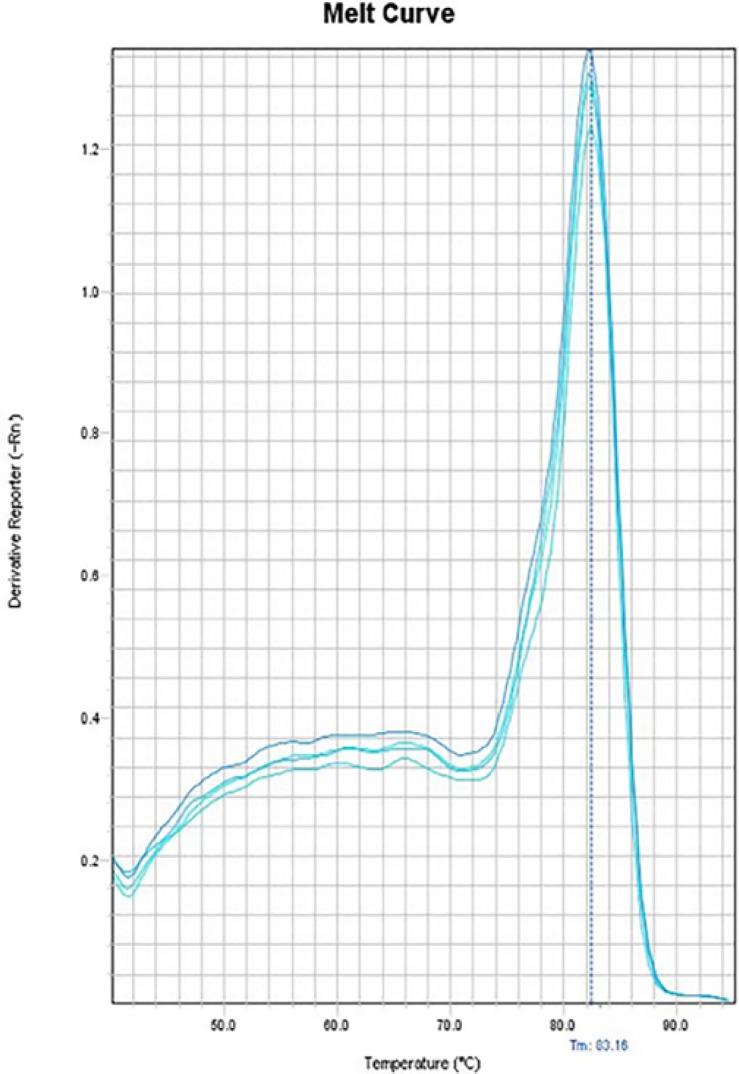
Melting curve of GAPDH gene

**Figure 13:**
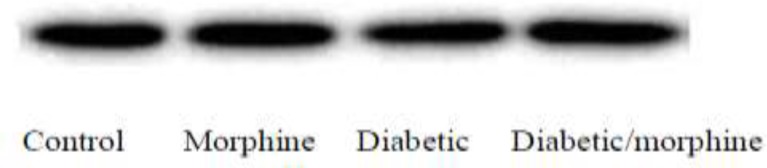
GAPDH expression, iNOS bands were not detected

Therefore, tolerance to morphine in the diabetic morphine tolerated group was occurred earlier than the non-diabetic group (on day 5 versus day 7) as shown in Figure 2 and 3.

In figure four we can see that the pain threshold in diabetic morphine tolerated group is significantly increased on day 3 but decreased on day 5 compared to non-diabetic morphine tolerated animals (Figure 4). 

After the establishment of tolerance in morphine tolerated groups (day 7) Naloxone induced withdrawal signs in the diabetic morphine tolerated group was significantly decreased compared to the non-diabetic morphine tolerated group (Figure 5).

*Effects of experimental diabetes and morphine tolerance on CAT-2 and iNOS expression*

In the molecular part of the study, the real time PCR data showed that there were no significant differences in CAT-2 and iNOS gene expressions between morphine tolerated and control groups in the brain and spinal cord tissues (Figure 6). Likewise, this comparison between diabetic and control groups showed no significant differences (Figure 7).

However, iNOS gene expression in the spinal cord tissues increased in the diabetic morphine tolerated rats compared to all other groups (Figure 8, 9). 

In the melting curves of CAT-2 (Figure 10), iNOS (Figure 11) and also the house-keeping gene, GAPDH (Figure 12) only one major peak is seen which means that our samples are pure.

In completing the study, iNOS protein was not detected by western blotting while GAPDH protein was clearly seen (Figure13). 

## Discussion

Our study showed that there was a significant increase in PWL in diabetic rats compared to control group. Our data also indicated that tolerance to morphine in the diabetic group was occurred prior to the non-diabetic group. On the other hand, withdrawal symptoms in diabetic rats were significantly less than that in non-diabetic animals. 

Similarly Leedom and Raz showed an increase in pain threshold in early diabetic state and they concluded that it may be due to the increase in compensatory secretion of endogenous opioid peptides such as β-endorphin (18, 19).

Joharchi and Jorjani in 2007 observed that analgesia, or increase in hot plate latency time, is significantly higher in diabetic rats compared to non-diabetic group in single dose administration of morphine sulfate, but tolerance to morphine analgesia in diabetic group is more than non diabetic animals in acute pain. (25).Similarly, some other studies revealed that acute hyperglycemia significantly decreases the hot plate latency time in diabetic rats in comparison to control non-diabetic animals (17, 33).

This study also showed that there was no significant difference between diabetic and control groups during the last days of our experiment which is identical to our previous study (25) and to the study of Morley *et al.*(17)

After tolerance to morphine, our results showed that in the first days of diabetes, morphine could increase nociception threshold but decreased during the last days of the experiment which is in consistent to the previous studies indicating that the potency of morphine is decreased in the diabetic state (18-20, 23). 

However, in our previous study, the anti-nociceptive effect of morphine decreased significantly compared to the non-diabetic state in chronic treatment (34). Decreasing in the morphine effect in chronic state may be due to the tolerance to morphine as seen in the last days of this study.

On the other hand, in our study, tolerance to morphine occurred in diabetic animals prior to non-diabetic rats. It seems that diabetes has an important role in morphine tolerance and also in pain perception. 

We also found that diabetes can significantly decrease withdrawal symptoms caused by morphine deprivation. This phenomenon may be due to the lower effect of morphine in diabetes and hyperglycemia. It has been also shown in our other study that withdrawal syndrome significantly decreases in diabetic rats (34). Kamei and Ohsawa reported that the central noradrenergic system may be responsible for the change in behavior of diabetic rats with withdrawal syndrome (35). Another study has also shown that hyperglycemia suppresses morphine withdrawal symptoms (36). 

In our molecular study, we have found that in spinal cord tissues, expression of iNOS gene was significantly increased in diabetic morphine tolerated rats compared to diabetic group and also compared to non-diabetic morphine tolerated animals. But there was no significant difference between diabetic, morphine tolerated, and control groups. Many mechanisms are involved in morphine tolerance and neuropathic pain (37). 

As we have suggested previously one of these mechanisms may be the involvement of Nitric Oxide (25). NO has a significant role in nociception, inflammation, neuropathic pain, and morphine tolerance (11, 12, 38-42). It has been shown that pretreatment with iNOS inhibitor decreases the development of morphine tolerance and dependence in mice (43). We have shown that there is an increase in urinary nitrite concentration in diabetic state and also in morphine tolerance which suggests that the increase of morphine tolerance in diabetic rats may be due to a rise in nitric oxide (25). Another study in 2013 showed that iNOS mRNA expression is increased in mice brain after repeated injection of morphine (44).

Considering previous studies and also our data, we can conclude that morphine and diabetes together enhance nitric oxide synthesis and this issue may be due to a significant increase in expression of iNOS in the spinal cord level, while supraspinal is not affected. It seems that, there may be a crosstalk between these two processes, the morphine tolerance and the diabetes, in elevating the amount of NO in spinal cord via the elevation of iNOS.

Our study on the expression of CAT-2 showed that there was no significant difference in expression of this transporter between any experimental groups. This finding may indicate that this transporter has no role in morphine tolerance and diabetes. Moreover, there are no enough reports about the role of CAT-2 expression in these processes. Based on the above mentioned rationale, it is probable that transporters may have no role in increasing the nitric oxide, but it seems that the iNOS plays a main role in the spinal cord in this regard. Thus, more comprehensive studies are needed to clarify the exact mechanisms.

The other conflicting result that we have obtained is that iNOS genes are not translated into proteins in any group, or we could not detect it by western blot technique. This means that longer studies may be needed in order to establish a chronic state and protein synthesis.

## Conclusion

It seems that increase of nitric oxide in diabetes and also in morphine tolerance may be due to the increase in expression of induced nitric oxide synthase in the spinal cord of male rats. 

## Conflict of interests

There is no conflict of interests.
